# Spindle Cell Variant of Ameloblastic Carcinoma: Another Example in a Japanese Male

**DOI:** 10.1155/2023/8755637

**Published:** 2023-03-16

**Authors:** Hiroshi Harada, Akio Tanaka, Masatomo Kimura, Shinsuke Ieda, Takeshi Wada, Tatsushi Matsumura, Akira Kurose

**Affiliations:** ^1^Department of Anatomic Pathology, Hirosaki University Graduate School of Medicine, Hirosaki, Japan; ^2^Department of Diagnostic Pathology, Hashimoto Municipal Hospital, Hashimoto, Japan; ^3^Department of Oral and Maxillofacial Surgery, Hashimoto Municipal Hospital, Hashimoto, Japan; ^4^Department of Oral and Maxillofacial Surgery, Wakayama Medical University, Wakayama, Japan

## Abstract

Spindle cell variant of ameloblastic carcinoma (SpCAC) is a rare subtype of ameloblastic carcinoma. Herein, we describe an additional case of SpCAC of the mandible of a 76-year-old Japanese male. We discuss diagnostic problems we encountered in this case, focusing on unusual expression of myogenic/myoepithelial markers, such as smooth muscle actin and calponin.

## 1. Introduction

Spindle cell variant of ameloblastic carcinoma (SpCAC) is a rare subtype of ameloblastic carcinoma [1–3]. According to the current 4th edition of the World Health Organization classification of odontogenic tumors [1], it has been reported that some examples of SpCAC have followed an aggressive course.

Herein, we describe an additional case of SpCAC arising in the mandible and compare the clinicopathological features presented in the literature.

## 2. Case Report

**Figure 1:**
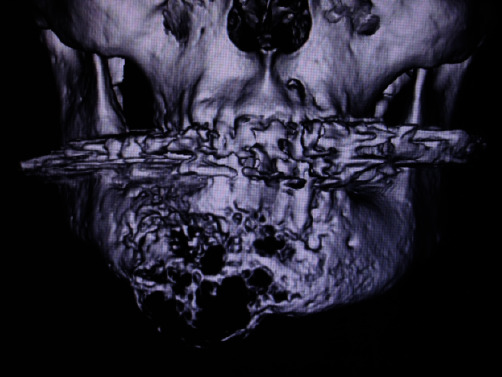
3D-CT showing wide bone destruction of the right anterior mandible, suggesting tumor extension beneath the mucosa and skin from the cortical bone. Small bone fragments have been pushed away to the outside.

The patient was a 76-year-old Japanese male with history of hypertension, type 2 diabetes, diabetic nephropathy, arteriosclerosis, and myocardial infarction, in addition to abdominal aortic artificial blood vessel replacement. He had been aware of pain in his right mandible for approximately 10 years. He had his second premolar extracted at a local dental office, but was referred to a regional general hospital because of poor healing of the extraction socket. A biopsy from the extraction socket was interpreted as malignancy, but was not informative for a specific diagnosis. Although subsequent treatment was prepended by recurrent myocardial infarction, he underwent radiation therapy of total dose of 60 Gy after waiting for 4 months for his general condition to recover. Thereafter, he requested conservative treatments, and outpatient observation was continued. However, the tumor grew further, and three-dimensional computed tomography (3D-CT) revealed wide bone destruction of the right anterior mandible along with tumor extension beneath the mucosa and skin from the cortical bone (Figure 1). Then, partial resection of the tumor was performed for the purpose of volume reduction. He was subsequently transferred to another hospital for treatment of diabetic nephropathy and died of renal failure approximately 1 year after his first visit. Before the transfer, metastasis to the vertebra was revealed by X-ray and CT, which was histologically confirmed by a biopsy performed by orthopedic surgeons. Autopsy was not performed. However, no other primary cancer was found during the clinical course, despite thorough examination including positron emission tomography.

## 3. Pathological Findings

**Figure 2:**
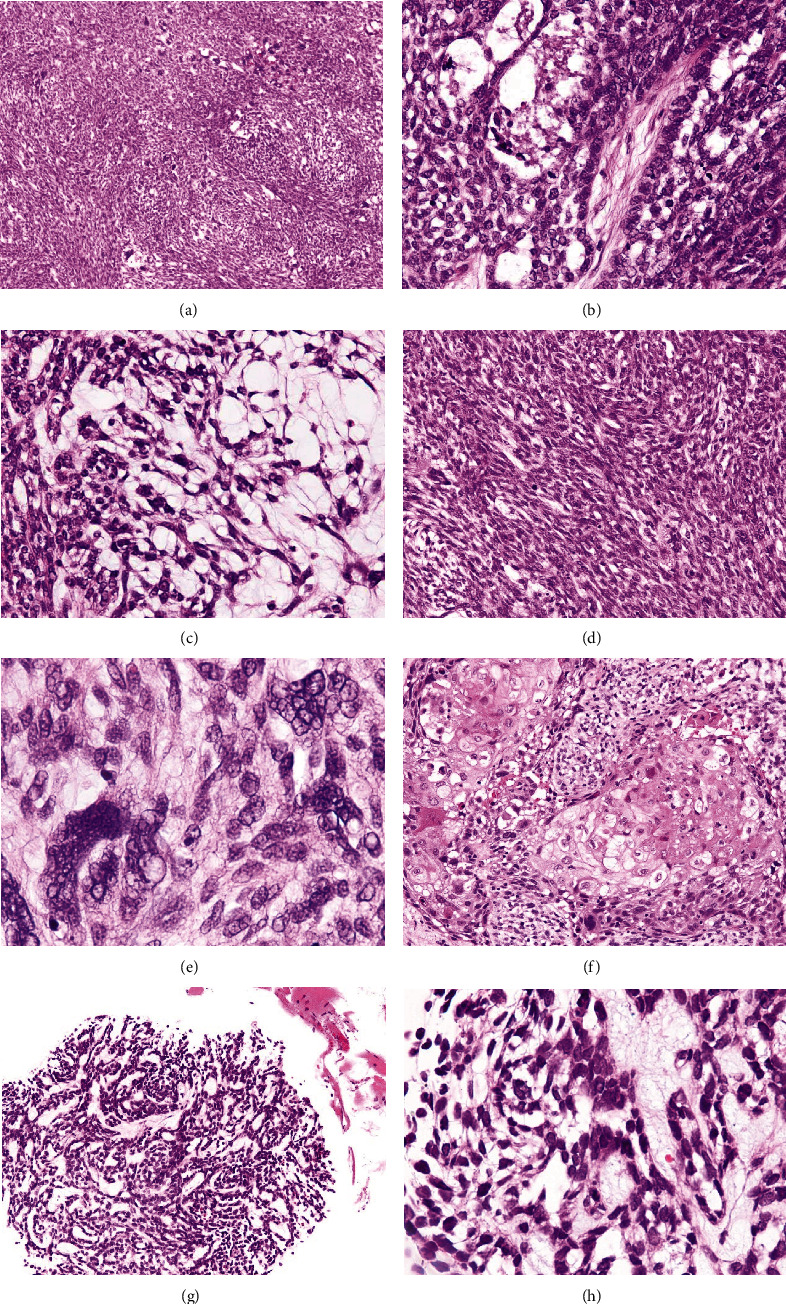
Light microscopic findings. Spindle cells dominate within the tumor and are associated with bizarre giant/multinucleated cells (a). Basaloid tumor cells form fusing nests with peripheral nuclear palisading (b) and characteristic cell dissociation reminiscent of enamel pulp (c). Spindle cells showing a storiform-like pattern (d) and bizarre giant/multinucleated cells (e). Small keratinizing foci are apparent (f). The metastatic tumor of the vertebra appearing along with surrounding soft tissue consisting of bland basaloid cells (g), and accompanied by ambiguous pseudolumina as well as intercellular dissociation of stellate cells (h).

Histopathologically, the tumor exhibited submucosal infiltration of basaloid cells along with predominant spindle cell proliferation (Figure 2(a)). Basaloid cells formed fusing nests with peripheral nuclear palisading (Figure 2(b)) and characteristic cell dissociation reminiscent of stellate reticulum (Figure 2(c)), while spindle cells were spread showing sarcomatoid fascicular arrangement (Figure 2(d)), and were associated with numerous bizarre giant/multinucleated cells (Figure 2(e)). Mitotic figures were readily recognized. Furthermore, small keratinizing foci were sporadically encountered (Figure 2(f)). However, mucosal dysplasia or carcinoma in situ was not noted, as far as the observation was possible.

A shallow biopsy from the vertebra revealed a small amount of metastatic tumor tissue consisting of bland basaloid cells along with surrounding soft tissue (Figure 2(g)), which was accompanied by ambiguous pseudolumina and intercellular dissociation of stellate cells identical to that of the mandibular tumor (Figure 2(h)). Spindle cell proliferation was not observed in the specimen.

**Figure 3:**
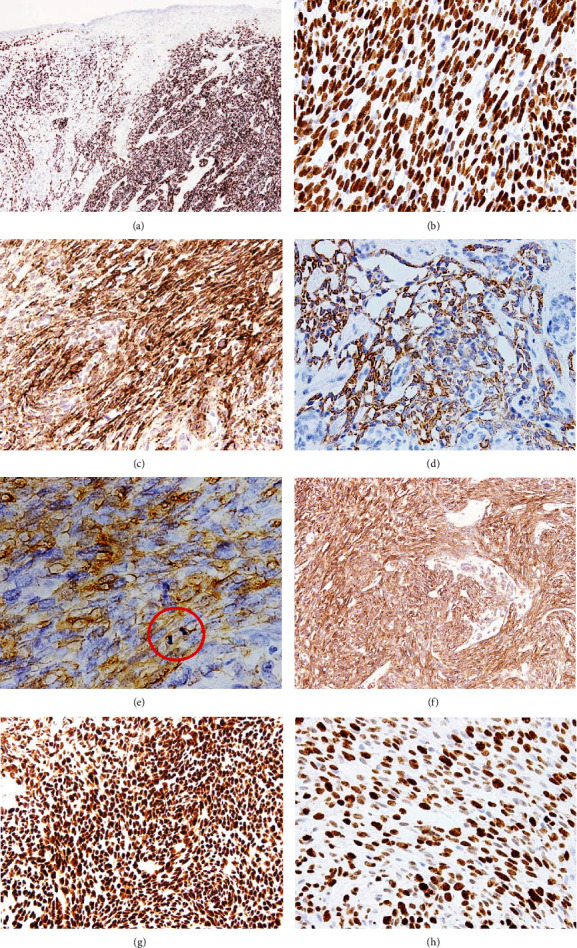
Immunohistochemistry. Tumor cells are diffusely immunoreactive for p40 widely spreading beneath the eroded mucosa (a). Spindle cells in a fascicular arrangement also indicate p63 immunoreactivity (b). Spindle cells are also immunoreactive for CK5/6 (c). A better differentiated area including the portion reminiscent of enamel pulp shows a positive reaction for CK19 (d). Spindle cells show a patchy reaction for aSMA (e). Brisk mitotic figures are evident in the spindle cell fascicle (red circle). Similarly, immunoreactivity for calponin is observed (f). Almost all tumor cells are labelled with p53 (g), and the vast majority with Ki-67 (h).

Immunohistochemically, the tumor cells were diffusely positive for p40 (Figure 3(a)) and p63 (Figure 3(b)), and positive for vimentin, AE1/AE3, CK5/6 (Figure 3(c)), and CK19 (Figure 3(d)) in addition to alpha-smooth muscle actin (aSMA; Figure 3(e)) and calponin (Figure 3(f)). CK7, S100 protein, glial fibrillary acidic protein, SOX10, WT1 (6F-H2), desmin, h-caldesmon, myogenin, and CD99 were negative. p53 was diffusely and intensely positive in the tumor cells (Figure 3(g)), and Ki-67 (MIB1) labelling index reached a maximum of 50% (Figure 3(h)).

## 4. Discussion

Our findings in this study are consistent with SpCAC, but the immunoreactivity for aSMA as well as calponin is a noteworthy feature. This variant is considered to have caused sarcomatoid changes in ameloblastic carcinoma [3], but the odontogenic epithelium and the mucosal epithelium of the upper aerodigestive tract including the oral cavity are not developmentally separated, and odontogenic tumors essentially have similar cellular properties to squamous neoplasms [4]. Furthermore, spindle cell (squamous) carcinomas, which are sarcomatoid carcinomas of the head and neck mucosa, sometimes show positive reactivity for myogenic markers, such as actin or desmin [5, 6]. Thus, actin immunoreactivity of the present case caused no contradiction. Also, co-expression of aSMA and calponin is unsurprising, because a close relationship between actin and calponin in their structural and functional aspects is already known.

Among bone and soft tissue sarcomas, expression of epithelial markers is not restricted to synovial sarcoma or epithelioid sarcoma. In fact, epithelioid osteosarcoma may express either epithelial membrane antigen or cytokeratin also in the jawbone [7]; however, epithelioid osteosarcoma differs to SpCAC at the point of predominantly epithelioid morphology. The present tumor did not include true glandular element as observed in synovial sarcoma. Even if rare examples of mandibular synovial sarcoma have been described, SpCAC is more likely to be considered in the mandible.

Although such exceptions actually exist, in general, diffuse and intense immunoreactivity for p40 and p63 may help distinguish SpCAC from true sarcomas. CK19, a low molecular weight cytokeratin, which is also used as a marker for odontogenic epithelium, could be still suggestive of odontogenic origin, although this is not always specific [3, 4].

Furthermore, gnathic osteosarcomas are known to occasionally express S100 protein or actin [8], and of course, prudent identification of osteoid formation is required, which was completely lacking in this case. Expression of actin and calponin, also myoepithelial markers, in ameloblastic carcinoma can be a potential diagnostic pitfall. In fact, exceptional cases of intraosseous myoepithelial tumors of the jawbone have been described in the past and recently [9], basaloid cellular morphology itself is reminiscent of some salivary gland-type tumors including adenoid cystic carcinoma forming cribriform or pseudoglandular structures [2]. However, appropriate judgements can be made on the basis of an accurate and comprehensive understanding of every condition including the expression of other myoepithelial markers.

In the recent case reported by Takahata et al. [3], in which the current primary author (HH) was involved for histopathological analysis, eosinophilic granular cells, which are morphologically identical to those in soft tissue granular cell tumors, were quite unique and were helpful in the recognition of ameloblastomatous features in their case. These cells are not always present in ameloblastoma or ameloblastic carcinoma, and were absent in our case, but small keratinizing foci were identified instead.

A distant metastasis of concern appeared in the vertebra at the terminal phase in the present case, but the distribution to the skull base, both lungs, and the pleura in the case of Takahata et al. [3] should be regarded as exceptional, and it is presumed that one of the causes of widely spread metastases during the course of the year could be repeated surgical invasion of the mandibular bone bearing the tumor.

In our case, the metastatic tumor in the vertebra revealed almost a pure basaloid population lacking spindle cell proliferation in the specimen, although the overall histology was unknown. Pathologists sometimes experience a tumor whose image differs histologically from that of the primary tumor at the metastasis site. The most likely explanation is a change in the microenvironment at the metastasis site or irradiation involvement, which we discussed in our previous report [10]. In the current case, it should be noted that the patient underwent radiation therapy for the primary tumor of the mandible. Alternatively, another possibility may be that a benign ameloblastoma underwent malignant transformation after having produced a bone metastasis to the vertebra.

## 5. Conclusion

SpCAC is extremely uncommon, but can be an aggressive and even lethal jawbone neoplasm that should be distinguished from sarcomatous lesions of the bone and soft tissue. Whether the clinical course is long-term or not, it is necessary to strictly recognize that SpCAC can metastasize, even though ameloblastoma rarely metastasizes despite its benign histology.

## Data Availability

The data used to support the findings of this study are available from the corresponding author upon request.
